# Locating helicopter emergency medical service bases to optimise population coverage versus average response time

**DOI:** 10.1186/s12873-017-0142-5

**Published:** 2017-10-16

**Authors:** Alan A. Garner, Pieter L. van den Berg

**Affiliations:** 1 CareFlight, Northmead, NSW Australia; 2 Rotterdam School of Management, Rotterdam, The Netherlands

**Keywords:** Helicopter, Bases, Modelling, Optimization, Coverage, Response, Population

## Abstract

**Background:**

New South Wales (NSW), Australia has a network of multirole retrieval physician staffed helicopter emergency medical services (HEMS) with seven bases servicing a jurisdiction with population concentrated along the eastern seaboard. The aim of this study was to estimate optimal HEMS base locations within NSW using advanced mathematical modelling techniques.

**Methods:**

We used high resolution census population data for NSW from 2011 which divides the state into areas containing 200–800 people. Optimal HEMS base locations were estimated using the maximal covering location problem facility location optimization model and the average response time model, exploring the number of bases needed to cover various fractions of the population for a 45 min response time threshold or minimizing the overall average response time to all persons, both in green field scenarios and conditioning on the current base structure. We also developed a hybrid mathematical model where average response time was optimised based on minimum population coverage thresholds.

**Results:**

Seven bases could cover 98% of the population within 45mins when optimised for coverage or reach the entire population of the state within an average of 21mins if optimised for response time. Given the existing bases, adding two bases could either increase the 45 min coverage from 91% to 97% or decrease the average response time from 21mins to 19mins. Adding a single specialist prehospital rapid response HEMS to the area of greatest population concentration decreased the average state wide response time by 4mins. The optimum seven base hybrid model that was able to cover 97.75% of the population within 45mins, and all of the population in an average response time of 18 mins included the rapid response HEMS model.

**Conclusions:**

HEMS base locations can be optimised based on either percentage of the population covered, or average response time to the entire population. We have also demonstrated a hybrid technique that optimizes response time for a given number of bases and minimum defined threshold of population coverage. Addition of specialized rapid response HEMS services to a system of multirole retrieval HEMS may reduce overall average response times by improving access in large urban areas.

**Electronic supplementary material:**

The online version of this article (10.1186/s12873-017-0142-5) contains supplementary material, which is available to authorized users.

## Background

Helicopter Emergency Medical Services (HEMS) form an important component of the prehospital care system in many developed jurisdictions. HEMS can deliver advanced medical care to the scene of an incident, rapidly transport patients to specialist centres or provide a rescue capability that may not be possible or timely by surface vehicle. HEMS generally provide a higher level of clinical care than is available by road EMS systems as helicopters enable a single, more highly trained team to cover a large geographical area. HEMS are also expensive resources that require optimal base locations to maximise both clinical effectiveness and operational efficiency. A mathematical model such as the Maximal Covering Location Problem (MCLP) [1] can be utilised to determine both the optimum location of a defined number of bases to produce the highest percentage of population coverage, or the minimum number of bases that would be required to meet a defined threshold percentage. The MCLP model has found a wide range of applications including in health sciences and emergency services [2, 3]. It is also possible to optimise base locations based on other criteria such as minimising average response time to the entire population using an Average Response Time Model (ARTM) which may produce different optimal base locations to the MCLP model. This model was introduced by ReVelle [4] as the p-median problem and was, for example, applied to emergency services by Dzator [5]. Using the State of New South Wales, Australia as an example this study seeks to mathematically determine optimum HEMS base locations comparing models optimised for population coverage versus optimisation to minimise average response times, as well as exploring a hybrid optimisation model.

An additional element that we sought to explore was the effect of HEMS configurations on optimisation models. In many high density European countries HEMS (E-HEMS) roles are specialised with separate services conducting scene response, interfacility transfer and search, rescue and hoisting (SAR) roles. In jurisdictions such as Australia however there are a small number of large urban areas but population density is otherwise very low with large transport distances. The Australian HEMS model utilises multirole retrieval HEMS (MR-HEMS) services that have a broad scope of operations including offshore and mountain hoist rescue, specialised interfacility transports such as ECMO, IABP and neonatal transfers as well as scene response. Although previous studies have demonstrated significantly faster response times in Sydney for an E-HEMS type service [6], the dispatch system currently does not recognise the difference in response capabilities between this and the MR-HEMS [7]. A secondary objective of this study was therefore to model the response time effect of an E-HEMS type service in the large urban area of Sydney, Australia when superimposed upon a system otherwise exclusively operating MR-HEMS.

We therefore explore the mathematically optimal locations of MR-HEMS bases in NSW comparing the MCLP and ARTM and a proposed hybrid model. Using detailed population density data for the whole of NSW, we fit MCLP and ARTM to explore optimal base structures. We performed both “green field” analyses, assuming a hypothetical situation with no current MR-HEMS bases, and optimisation conditioned on the current bases, in order to explore whether improvements to the existing base structure could be achieved by moving or adding a few select bases. We also explore the effects of a specialist E-HEMS type service when overlayed on the MR-HEMS network in NSW.

## Methods

### Setting

NSW currently has a system of nine MR-HEMS services operating from seven bases 24 h a day to cover a population distributed over more than 809,000 km^2^. The population is however mostly urban and coastal, being concentrated in Sydney and Newcastle. The Australian Capital Territory (ACT) is not administratively part of NSW, but NSW Government funds an ACT based MR-HEMS to provide services to NSW residents in the south east of the State. Hence, the ACT based MR-HEMS functionally forms part of the NSW retrieval system and is part of the seven base network. In addition, a specialist rapid response prehospital helicopter service (E-HEMS model as typically utilised in densely populated European countries such as Germany and the Netherlands) operates from a hospital site near to the demographic centre of Sydney (CareFlight Rapid Response Helicopter - CRRH). This service is not part of the MR-HEMS network and does not perform interhospital transfers or hoist rescue, providing a dedicated prehospital service only operating exclusively in the area up to 60 nm (111 km) of its base near the demographic centre of Sydney. The area covered by the CRRH includes 70% of the total NSW population. It operates 12 h a day year round (rather than daylight hours specifically) due to funding limitations. Both MR-HEMS and the CRRH are tasked by a central dispatch system operated by NSW Ambulance [7] utilising the same criteria. Road ambulances are routinely dispatched in parallel to all HEMS responses.

The most recent publicly available data from 2011 indicates that there were 3339 pre-hospital and inter-hospital HEMS missions across NSW including those performed by the ACT and CRRH services [8]. In 2015, there were 3970 significant trauma patients (Injury Severity Score greater than twelve) in NSW, with 28.9% of injuries sustained in rural areas [9]. Across NSW, 15.4% of all severe trauma cases were transported by helicopter, but in rural areas this was 39.1% of cases.

### Data sources

The most recent Australian census from which detailed population data is available was conducted in 2011 when the population of NSW was 6.9 million people. The finest resolution of data available from the census is Statistical Area 1 (SA1) level data [10]. SA1s generally have a population of 200 to 800 persons, and an average population of about 400 persons. NSW contains 17,891 SA1s. The population of each SA1 was assumed to be located at the geographic centrum of the SA1 for modelling purposes. Lord Howe Island although administratively part of NSW was excluded from the modelling as it represents a single SA1 unit with a 2011 population of only 360 people. It is 600 km off shore and is serviced exclusively by fixed wing air ambulances.

### Model assumptions

Contracted times for highest priority missions during daylight hours for MR-HEMS bases is a median of 10 min from notification to airborne. This aligns closely with the actual helicopter response times reported across all NSW MR-HEMS bases [11]. The CRRH service does not have a contracted response time but is airborne a median of 4 min from beginning of the tasking phone call. For modelling purposes, we assumed the 10 min contracted time from notification to airborne for all bases apart from the CRRH where the actual median of 4 min was used.

The most recent round of tendering for MR-HEMS in NSW moved all bases to airport locations in anticipation of higher helicopter performance standards being imposed by the Australian civil aviation regulator. The ACT MR-HEMS and the CRRH, however, continue to operate from non-airport locations. Possible base locations used in the modelling therefore were all of the 233 airfields in NSW plus the existing ACT and CRRH bases. The base locations used for the status quo model are the locations established at airports under the current contractual arrangements in addition to the ACT and CRRH bases.

Helicopter ground speed was assumed to be 250 km/h as an overall mean although this will vary with wind direction and strength. Although there is no mandated time to patient access in NSW, we have arbitrarily chosen 45 mins from activation to enable comparison with HEMS base location modelling studies from other jurisdictions with similar population size and densities such as Norway [12]. HEMS coverage within 15mins as is achieved in densely populated European countries such as Germany which has a population density of over 230 persons per square kilometre is not financially achievable in a jurisdiction with the population density of NSW which has nine persons per square kilometre. The Norwegian target of 45mins was therefore considered an appropriate international benchmark for comparison with NSW as it is both a high income jurisdiction and has very similar population density.

### Modelling methods

Optimal base locations were determined by approaching the question as an MCLP or by the ART model. The MCLP model maximises the number of SA1s covered by at least one MR-HEMS, weighted by the number of inhabitants in each SA1 location. That is, it maximises the population covered within a desired service distance, or time, by optimal allocation of a predefined fixed number of base facilities. Conversely, the model can be used to determine the least number of bases needed in order to guarantee a certain coverage of the population.

The MCLP model places one HEMS at each base location, assuming that each HEMS is always available. While in practice, this might be overly optimistic, the model was chosen as it represents a best-case scenario. The number and location of bases is the minimum needed in order to achieve a given population coverage within the defined time threshold. The travel times, including a 10 min fixed pre-flight preparation time (4 min for the CRRH), from all potential base locations to all demand locations was then calculated, and optimal base locations determined.

As with the MCLP model, the ARTM also assumes that each HEMS is always available. In this model, the goal is to minimise the average response time to the entire population, rather than optimise the coverage within a predefined time threshold. Consequently, the model benefits from response times significantly shorter than the threshold if bases are located in proximity to areas of high population density. The model is therefore likely to result in different optimal base configurations that have shorter response times in highly populated areas, but have lower overall coverage.Both models are Integer Linear Programming models that can be solved to optimality by solvers such as CPLEX and Gurobi that use advanced mathematical techniques to guarantee optimality. This means that no alternative solution exists that performs better on the objective used in the model.

To explore the practical consequences of various base locations using the MCLP, we calculated the number of bases needed to cover various percentages of the population for the threshold time of 45 min from activation to patient contact for the MR-HEMS system. This yields the optimal base locations for MCLP using the chosen set of parameter values. For the ART model we calculated, for the same number of bases, the base location configuration which results in the lowest average time to patient contact across NSW. For both optimisation models, we first computed the optimal base locations assuming no current bases existed, so-called “green field” analysis. It is acknowledged that such an analysis is unlikely to be practicably feasible, as rebuilding already existing infrastructure to optimise system performance would be costly. We thus also performed conditional optimisation, i.e., given the existing 7 bases in NSW, what would be the possible additional gain of moving or adding one or two bases, still optimised for performance. The CRRH base was evaluated only for the ARTM as it is based just 13 km from the MR-HEMS base in Sydney and only operates within 60 Nm of its base. The CRRH radius of operation is therefore entirely within the 45 min response circle of the closest MR-HEMS base and therefore cannot provide additional population coverage in the MCLP model. Finally, an optimised hybrid green field model was also constructed where the average response time was optimised for a defined minimum threshold level of population coverage.

The models are implemented in Java and solved with IBM ILOG CPLEX Optimization Studio (CPLEX 12.6.2).

## Results

**Fig. 1:**
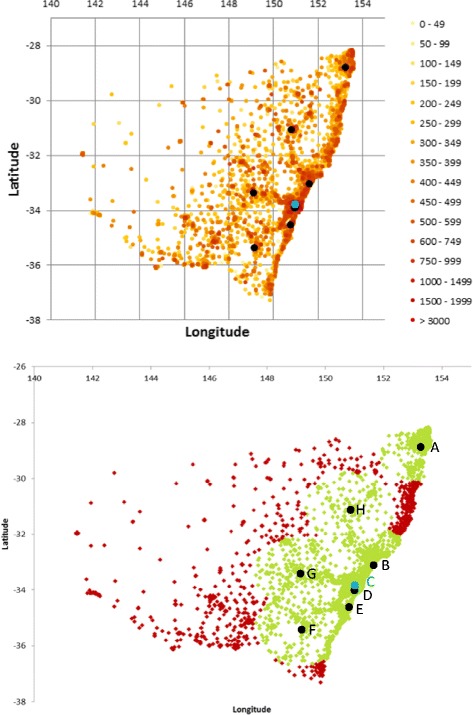
The diagram at the top shows the population density of NSW as per the 2011 census data with the current MR-HEMS base locations in black and the CRRH E-HEMS base in blue. The diagram below indicates in green the population that can be reached within 45mins of activation from all existing base locations at A. Lismore, B. Lake Macquarie Airport (Newcastle), C. Westmead (blue), D. Bankstown Airport, E. Wollongong, F. Canberra, G. Orange, and H. Tamworth

The population density of NSW is represented in Fig. 1 where colour dots are based on the number of inhabitants in a statistical area. The current MR-HEMS and CRRH base locations are superimposed. Fig. 1 also demonstrates the performance of the current base locations in providing coverage within the 45 min threshold. 91.10% of the population can be reached within 45mins of activation and the average state wide response time without the CRRH is 21.21 min.

### Optimal green field base locations for the MR-HEMS services

**Fig. 2:**
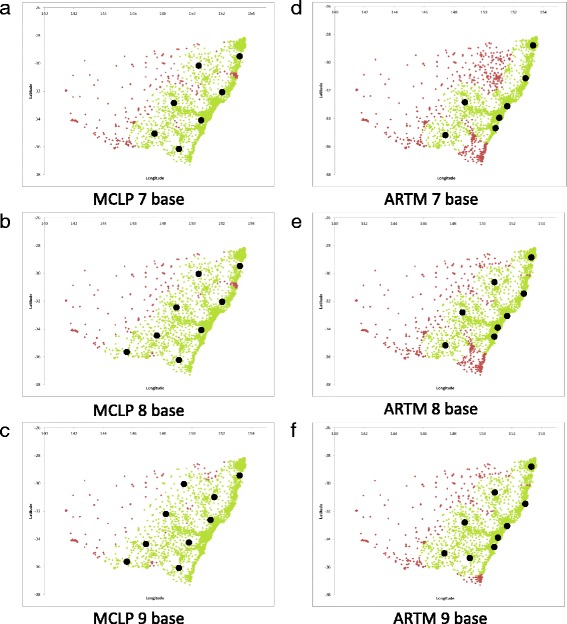
Greenfield MR-HEMS base locations for the MCLP and ARTM methods. **a,b** and **c** are the optimal MCLP solutions for 7, 8 and 9 bases respectively whereas **d**, **e** and **f** are optimal ARTM solutions for 7, 8 and 9 bases respectively

Table 1 and Fig. 2 detail the system performance when 7, 8 or 9 green field MR-HEMS base options are calculated. In the optimised coverage (MCLP) model, the average response time increases as base locations move away from the densely populated coastal strip although nine bases are able to cover 99.15% of the population.

**Table 1:** Population coverage and average response times with green field MR-HEMS base modelling by either the MCLP or ARTM

Number of modelled bases	Population covered	% population covered	Average response time (min)
*Greenfield locations optimised for population coverage (MCLP)*			
7	6,768,758	98.03%	27.22
8	6,819,726	98.77%	27.07
9	6,846,221	99.15%	36.11
*Greenfield locations optimised for average response time (ARTM)*			
7	6,510,149	94.29%	20.39
8	6,624,819	95.95%	19.69
9	6,740,249	97.62%	19.12

In the green field optimised average response time model (ARTM) moving from seven to nine bases decreases the average state wide response time by 1.27 min although population coverage also increases by more than 3% to 97.62%.

### Optimisation of fixed MR-HEMS base locations by moving or adding bases to existing locations

**Fig. 3:**
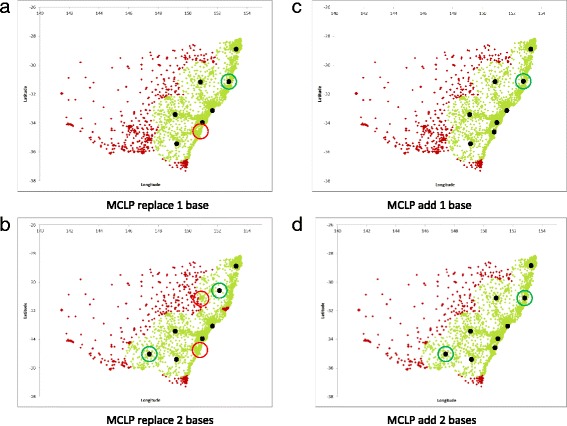
MCLP fixed MR-HEMS base solutions. **a** and **b** are the replace one base and replace 2 base solutions respectively whereas **c** and **d** are the add one base and add two base solutions respectively

**Fig. 4:**
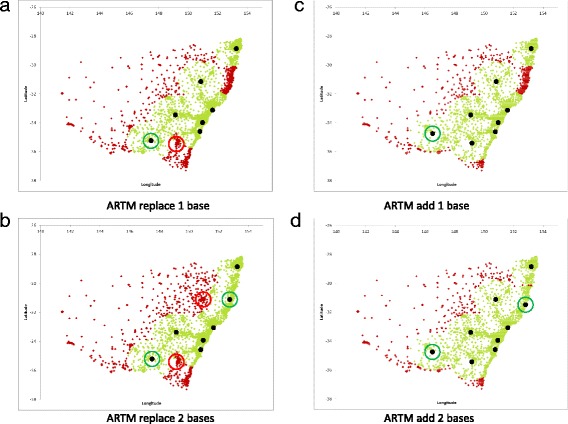
ARTM fixed MR-HEMS base solutions. **a** and **b** are the replace one base and replace 2 base solutions respectively whereas **c** and **d** are the add one base and add two base solutions respectively

Table 2 and Figs. 3 and 4 detail the effect of adding or moving one or two bases from the current seven MR-HEMS base locations. Results are presented for both the MCLP and ARTM.

**Table 2:** Population coverage and average response times with fixed MR-HEMS base modelling by either the MCLP or ARTM

Optimisation strategy	Population covered	% population covered	Average response time (min)
Current MR-HEMS base locations	6,289,847	91.10%	21.21
*Current MR-HEMS base locations optimised for coverage (MCLP)*			
Replace one base	6,519,110	94.42%	21.12
Replace two bases	6,650,701	96.32%	20.63
Add one base	6,519,110	94.42%	20.38
Add two bases	6,703,967	97.09%	19.15
*Current base locations optimised for response time (ARTM)*			
Replace one base	6,364,763	92.18%	20.66
Replace two bases	6,495,308	94.07%	20.44
Add one base	6,442,140	93.30%	20.08
Add two bases	6,668,560	96.58%	19.13

In the MCLP depicted in Fig. 3, the first base to be replaced is Wollongong which moves to Kempsey on the mid north coast. If two bases are moved, Tamworth moves east far enough to cover the Mid North Coast whilst Wollongong moves to Wagga Wagga. If bases are added the first base is at Kempsey and the second is at Wagga Wagga.

When average response time is optimised via the ARTM (Fig. 4), the first base relocated is Canberra which moves to Wagga Wagga and the second relocation is Tamworth to Kempsey. If bases are added the first is at Narrandera and the second is at Port Macquarie.

### Effects of adding the Sydney CRRH (E-HEMS model) base to average response times

As noted in the methods, addition of the CRRH to the 7 MR-HEMS bases makes no change to the percentage of population covered within 45 min as it operates exclusively within the 45 radius of action of the Sydney MR-HEMS base. The effect on average response time across the state, however, is a reduction by 3.65 min to 17.56 min. Within its area of operation the average response time was 14.94 min when only the MR-HEMS service was modelled which fell to 8.87 min when the CRRH (E-HEMS) was included. This is slightly more than the 6 min difference in response time as the CRRH base is located closer to the demographic centre of Sydney.

### Hybrid model

**Fig. 5:**
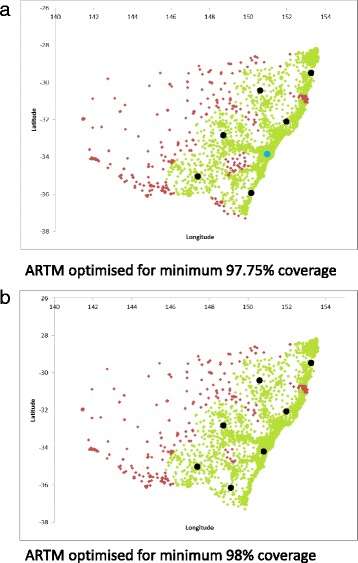
Hybrid solutions showing the optimal base locations to provide the shortest average response time for **a**, minimum 97.75% coverage and **b**, minimum 98% coverage. CRRH (E-HEMS) base location in blue. Scenario A represents the optimal trade-off between population coverage and average response time. In scenario B the population coverage increases by only 0.25% whilst the average response time jumps by 30% (8mins)

A hybrid greenfield model for seven HEMS bases including the CRRH base as a potential base location (hence benefiting from the potential inclusion of the rapid response capability of the CRRH E-HEMS model in the area of greatest population) was constructed for a range of coverage percentages between 95.94% (the coverage of the seven MR-HEMS base greenfield model when optimised for response time) and 98.03% (the coverage of the seven MR-HEMS base greenfield model when optimised for coverage). This is detailed in Table 3. There is an increase of nearly 8mins (30%) in the average response time due mostly to movement of the Sydney base away from the CRRH base location when the minimum population coverage moves from 97.75% to 98%. The locations of the bases for these two coverage scenarios are displayed in Fig. 5.

**Table 3:** Hybrid models designed to optimise response times with seven bases for a predetermined threshold percentage of population coverage

Model	% population covered	Average response time (min)
*Optimal ARTM*	95.94%	16.58
*≥ 96% coverage*	96.60%	16.61
*≥ 97% coverage*	97.12%	16.62
*≥ 97.25% coverage*	97.28%	16.73
*≥ 97.5% coverage*	97.54%	16.99
*≥ 97.75% coverage*	97.80%	17.93
*≥ 98% coverage*	98.00%	25.87
*Optimal MCLP*	98.03%	27.22

The optimal trade-off between population coverage and response time with seven bases is therefore a model covering just under 98% of the population in less than 45 min with an average response time to all inhabitants of the state in under 18 min. This consists of six MR-HEMS bases providing population coverage whilst benefiting from the addition of the E-HEMS (CRRH) base in Sydney to minimise average state wide response time. Optimal hybrid model base locations are at Westmead (E-HEMS) in Sydney with MR-HEMS bases at Gloucester, Yamba, Barraba, Yeoval, Wagga Wagga and Moruya.

## Discussion

As theorised, differing optimisation strategies for HEMS base placement produced varying base locations. In the green field modelling, an optimisation strategy that aimed to provide maximal population coverage moved bases away from the coast to provide some coverage inland whilst still covering coastal areas. This model resulted in longer average response times as the bases were positioned away from the largest population centres on the coast. However, an optimisation strategy that aimed at producing the lowest average response time positioned bases near the coast at the centres of maximal population density where the average response times were reduced by short distances to large numbers of people. The nine base MCLP green field model produced much longer average response times as there is not a base near to the population centre in Sydney. The existing base locations most closely resemble the green field ARTM locations although absence of HEMS coverage on the Mid North Coast is conspicuous when compared with all MCLP and ARTM green field models. Whether population coverage or response time is favoured in system design will generally be a political decision within a jurisdiction. We have however been able to demonstrate that a hybrid modelling strategy can be used to optimise average response times for a given level of population coverage which may provide an alternative middle ground strategy.

In the fixed base MR-HEMS modelling both the MCLP and ARTM moved or added bases to the Mid North Coast and Riverina (south-west of NSW) although the MCLP consistently added a base first to the Mid North Coast whereas the ARTM consistently prioritised a Riverina location for the first system reconfiguration. In the fixed base models both replacing and adding one base in the MCLP produced the same coverage result. This is because the Wollongong base behaves similarly to the CRRH base in decreasing overall average response time without contributing to 45 min response time coverage as its 45 min response circle lies entirely within the overlapping 45 min response circles of the Sydney and Canberra bases.

The current study uses advanced mathematical modelling techniques to predict the time that the HEMS team will be overhead the patient from the time that the service is notified. It does not take into account other variables that may affect the time to patient contact. For example, all HEMS services in NSW apart from Canberra and the CRRH now operate from airports. To achieve proposed aircraft performance requirements runway departures are required when the aircraft are fully fuelled, and air traffic control may create additional delays. When arriving at the scene the large MR-HEMS currently contracted in NSW may not be able to land as close to the scene as a smaller helicopter increasing the requirement for secondary ground transport to the incident scene. Factors such as these contributed to the large observed differences in time to patient contact in a previous study comparing the CRRH (E-HEMS model) to the MR-HEMS provided from the Bankstown Airport in Sydney [6]. That study also demonstrated that all other prehospital time intervals apart from transport time to hospital were also significantly faster in the CRRH despite similar levels of patient entrapment and clinical intervention. These differences were possibly due to the specialisation afforded by the restricted scope of E-HEMS operations compared with the MR-HEMS.

In the NSW system, prehospital helicopter teams always include a physician and represent the highest level of care available in the prehospital care system being used only in the most severe cases. Road ambulances are also always dispatched to an incident but are not a replacement for the physician teams. It can be postulated that it may be faster to provide physician teams in urban areas by road vehicle too but the previous study [6] also found that road response of physician teams from the Sydney base at Bankstown Airport was only faster than the CRRH to patients within 3 km of their base. Although optimisation models allowing for road transport over shorter distances have been developed [13], Sydney is a geographically large urban area of more than 5 million people with problematic road transportation infrastructure making timely road response from a single base to the entire urban area difficult. The constraints of landing large helicopters of the type used for MR-HEMS in NSW (AW139) in urban areas combined with slow road response capability due to traffic congestion may restrict access by specialised physician teams to patients in large densely populated areas unless more than one base is utilised. This study supports the idea that a small specialised E-HEMS operating in an urban area can significantly improve average response times at a state wide level by improving access to a large proportion of the overall state population.

There is an inevitable trade-off between population coverage and average response time when large portions of the population are situated in small areas of a jurisdiction that are not centrally located. NSW is an example of such a jurisdiction where the high-density areas occur at the coast on the edge of a very large service area. Equity of access to health care prioritises coverage over average response time. However, changes in average ambulance response times of as little as a minute are politically sensitive in Australia and access times therefore cannot be ignored in system planning. Although for ethical reasons it is impossible to definitively prove that time affects outcome, all EMS systems in the developed world are built around this premise including the NSW system. A hybrid design that optimises response times in high population urban areas and coverage in rural and remote areas may provide the best overall compromise. Specialised services such as the CRRH combined with the MR-HEMS services in NSW are an example of such a model in that the combination of base locations with differing response capabilities provides excellent average response times and as well as reasonable population coverage. The hybrid model used in this study sought to explore the inherent compromises between population coverage and response times at predetermined levels of coverage lying between the optimal ARTM and MCLP models. There is a distinct tipping point in average response time when the Sydney base is moved away from the CRRH base to improve overall coverage indicating that the CRRH is central to the performance of an optimised hybrid model.

This analysis has a number of inherent limitations. The model assumes that all helicopters are equally clinically capable whereas specialised medical teams such as neonatal and extracorporeal membrane oxygenation (ECMO) teams are only based at large urban locations. Therefore, even if a helicopter is available at a rural base, a helicopter from a central base with the specialist team will be utilised. The modelling also assumed that all airfields in NSW were appropriate HEMS base locations whereas rural towns such as Narrandera may not have local medical personnel of appropriate skill to support a HEMS service. Another limitation is that the model assumes that helicopters are always available at every base. In reality the higher the population is in a base’s catchment area the greater the probability that the service will be busy when required. Calculating the “busy fraction” for a base is not straightforward however and is likely to vary significantly between urban and rural bases. Models that take the busy fraction into account include the maximum expected covering location problem, [14] which maximises the weighted expected coverage of all demand locations while considering the probability that an emergency vehicle is available within the target response time, and in the maximum availability location problem [15]. In a jurisdiction such as NSW, the assumption that the busy fraction is similar for all demand zones is unrealistic, and zone-specific busy fractions would be required. Models that include the busy fraction are significantly more difficult to solve for regions of the size of NSW. Additionally, these models were developed for road ambulances that have significantly higher utilization. Increasingly large busy fractions would indicate the need to have more than one helicopter at a base location to maintain availability. The Bankstown base in Sydney currently has three helicopters reflecting the usage of these vehicles by centrally based specialist teams as well as the high demand levels associated with an urban location. Taking these factors into consideration the “ideal” solution modelled here could be further pragmatically refined by consideration of utilisation rates and specialist team requirements. It would be reasonable to conclude that Sydney also requires a MR-HEMS base in addition to an E-HEMS base for utilisation by specialist teams as the model developed here accounts only for prehospital response.

In the last 13 years two reviews of the NSW HEMS system including base locations have been commissioned by the NSW Government. The first review in 2004 identified the requirement for two new bases in addition to the existing seven MR-HEMS locations at Coffs Harbour and Wagga Wagga. Neither base was implemented prior to the subsequent review in 2012 (not publically released) which was based on the same 2011 census data used in this study. A Government Plan for NSW HEMS released in 2013 which was informed by the 2012 review does not plan any new HEMS bases however and makes no reference to the CRRH [9]. The advanced modelling techniques utilised in this study suggest that additional bases as previously suggested at Coffs Harbour and Wagga Wagga would increase both population coverage and improve average response times although the greatest gain in average state wide response time accrues from addition of the CRRH service in Sydney (calculated system performance with Coffs Harbour and Wagga Wagga bases added to the seven existing MR-HEMS bases is in included as Additional file 1).

## Conclusion

Advanced mathematical modelling techniques can be used to optimise MR-HEMS base locations based on either percentage of the population covered, or average response time to the entire population. We have also demonstrated a hybrid modelling technique that optimises response time for a given number of bases and minimum defined threshold of population coverage. Specialized E-HEMS services with different response characteristics can also be modelled and may reduce overall average response times for the entire jurisdiction by improving patient access times in large urban areas.

## Additional file

Additional file 1: MR-HEMS System performance with Coffs Harbour and Wagga Wagga MR-HEMS bases added. CRRH base not included in model. (TIFF 134 kb)

## Abbreviations

ACT: Australian Capital Territory
ARTM: Average response time model
CRRH: CareFlight Rapid Response Helicopter
ECMO: Extracorporeal membrane oxygenation
HEMS: Helicopter emergency medical services
MCLP: Maximal coverage location problem
NSW: New South Wales
SA1: Statistical area 1
