# Supplementary figures and images for: Locating helicopter emergency medical service bases to optimise population coverage versus average response time
Source: BMC Emerg Med. 2017 Oct 16;17:31. doi: 10.1186/s12873-017-0142-5 (PMC5644058; doi:10.1186/s12873-017-0142-5)

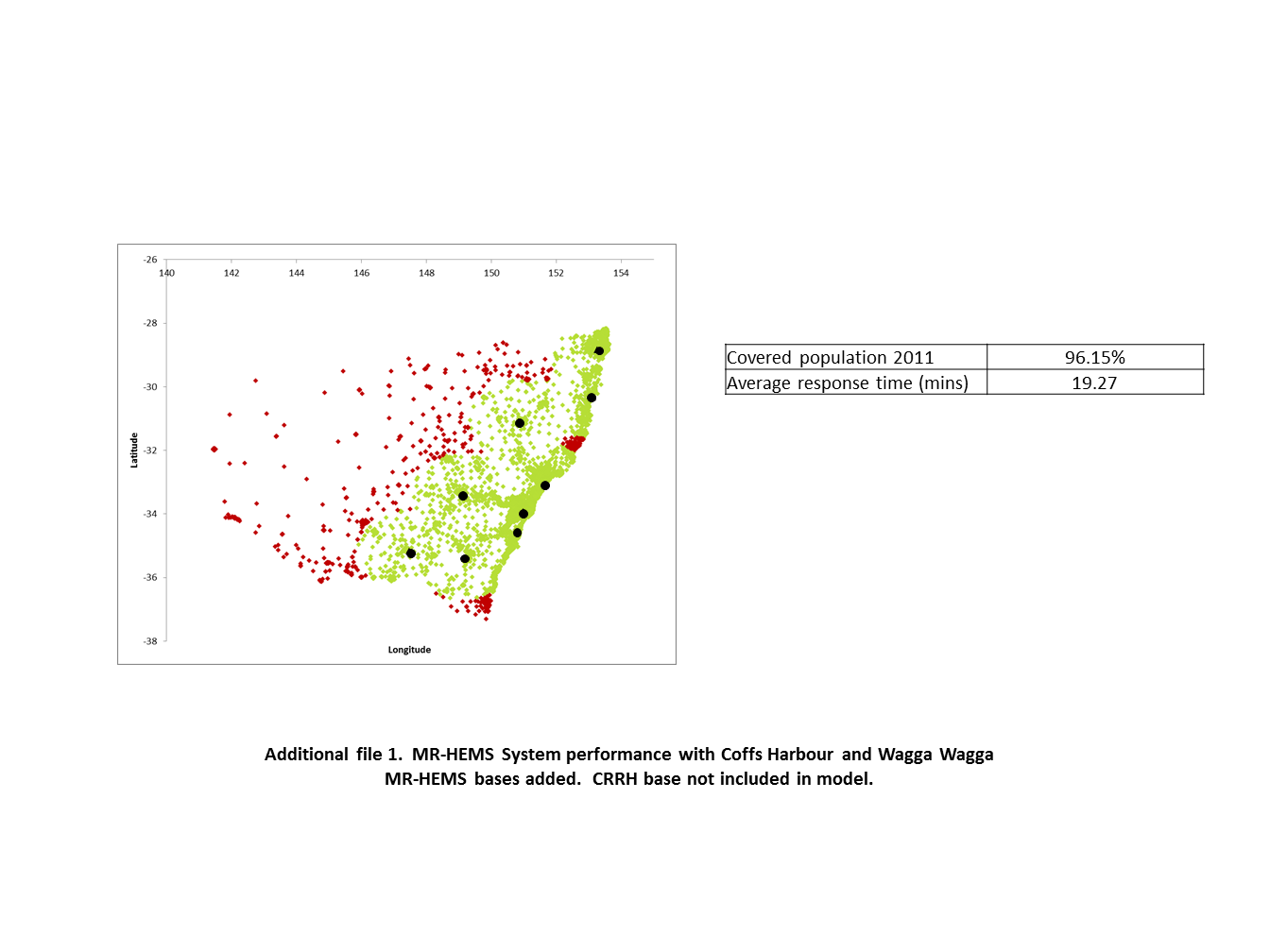

Supplement: Additional file 1: — MR-HEMS System performance with Coffs Harbour and Wagga Wagga MR-HEMS bases added. CRRH base not included in model. (TIFF 134 kb) [file 12873_2017_142_MOESM1_ESM.tif]
